# *GAS6-AS1*, a long noncoding RNA, functions as a key candidate gene in atrial fibrillation related stroke determined by ceRNA network analysis and WGCNA

**DOI:** 10.1186/s12920-023-01478-y

**Published:** 2023-03-09

**Authors:** Rui-bin Li, Xiao-hong Yang, Ji-dong Zhang, Wei Cui

**Affiliations:** Department of Cardiology, The Second Hospital of Hebei Medical University, No. 215 West Heping Road, Shijiazhuang, 050000 Hebei China

**Keywords:** Atrial fibrillation, Stroke, Competing endogenous RNA, Weighted gene co‑expression network analysis, Long non-coding RNA, *GAS6-AS1*

## Abstract

**Background:**

Stroke attributable to atrial fibrillation (AF related stroke, AFST) accounts for 13 ~ 26% of ischemic stroke. It has been found that AFST patients have a higher risk of disability and mortality than those without AF. Additionally, it’s still a great challenge to treat AFST patients because its exact mechanism at the molecular level remains unclear. Thus, it’s vital to investigate the mechanism of AFST and search for molecular targets of treatment. Long non-coding RNAs (lncRNAs) are related to the pathogenesis of various diseases. However, the role of lncRNAs in AFST remains unclear. In this study, AFST-related lncRNAs are explored using competing endogenous RNA (ceRNA) network analysis and weighted gene co-expression network analysis (WGCNA).

**Methods:**

GSE66724 and GSE58294 datasets were downloaded from GEO database. After data preprocessing and probe reannotation, differentially expressed lncRNAs (DELs) and differentially expressed mRNAs (DEMs) between AFST and AF samples were explored. Then, functional enrichment analysis and protein-protein interaction (PPI) network analysis of the DEMs were performed. At the meantime, ceRNA network analysis and WGCNA were performed to identify hub lncRNAs. The hub lncRNAs identified both by ceRNA network analysis and WGCNA were further validated by Comparative Toxicogenomics Database (CTD).

**Results:**

In all, 19 DELs and 317 DEMs were identified between the AFST and AF samples. Functional enrichment analysis suggested that the DEMs associated with AFST were mainly enriched in the activation of the immune response. Two lncRNAs which overlapped between the three lncRNAs identified by the ceRNA network analysis and the 28 lncRNAs identified by the WGCNA were screened as hub lncRNAs for further validation. Finally, lncRNA *GAS6-AS1* turned out to be associated with AFST by CTD validation.

**Conclusion:**

These findings suggested that low expression of *GAS6-AS1* might exert an essential role in AFST through downregulating its downstream target mRNAs *GOLGA8A* and *BACH2,* and *GAS6-AS1* might be a potential target for AFST therapy.

**Supplementary Information:**

The online version contains supplementary material available at 10.1186/s12920-023-01478-y.

## Introduction

Atrial fibrillation (AF), affecting 25% of adults worldwide, is the most common clinical tachyarrhythmia [1] and is independently associated with a two-fold risk of mortality [2, 3]. Stroke attributable to atrial fibrillation (AF related stroke, AFST) accounts for 13 ~ 26% of ischemic stroke [4], and this proportion increases with age [5]. AFST is characterized by a high percentage of early recurrent ischemic stroke [6] and hemorrhagic transformation (HT) in the days immediately following the index stroke [7]. AFST patients have a worse prognosis, including higher risk of disability and mortality, than those without AF [8]. Nowadays, a growing number of studies focus on preventing and intervening stroke in AF patients, however, the molecular mechanism of AFST is still not clearly understood, making its treatment a big challenge. Therefore, investigating the mechanism of AFST, as well as searching for the molecular targets for treatment, are of great clinical importance.

Long non-coding RNAs (lncRNAs) are a new kind of non-coding RNAs that lack of functional protein-coding ability [9], and are found of pronounced lower amounts than protein-coding genes. The function of lncRNAs in human transcription and epigenetics has been widely demonstrated [10]. Numerous research has shown that lncRNAs are related to various diseases, including cancer, heart failure, myocardial infarction and diabetes [11–14]. Despite these findings, the mechanism of lncRNAs in AFST remains unclear. According to the competing endogenous RNA (ceRNA) hypothesis, lncRNA can regulate messenger RNA (mRNA) expression as miRNA sponge [15]. By constructing disease-associated lncRNA-miRNA-mRNA regulatory ceRNA network, it is possible to identify disease-associated hub lncRNAs.

The weighted gene co-expression network analysis (WGCNA) is a relatively recent method to investigate the complex association between genes and clinical characteristics [16]. WGCNA can aggregate co-expressed genes into modules to identify disease-related hub genes. Co-expression modules associated with diseases can be constructed not only using mRNAs, but also miRNAs or lncRNAs [17, 18]. The method has been widely used to study plenty of diseases, including cancer [19], severe asthma [20], and proved to be an effective method to identify potential therapeutic molecular targets.

In this study, we aimed to identify potential hub lncRNAs associated with AFST using ceRNA network analysis and WGCNA.

## Materials and methods

**Fig. 1:**
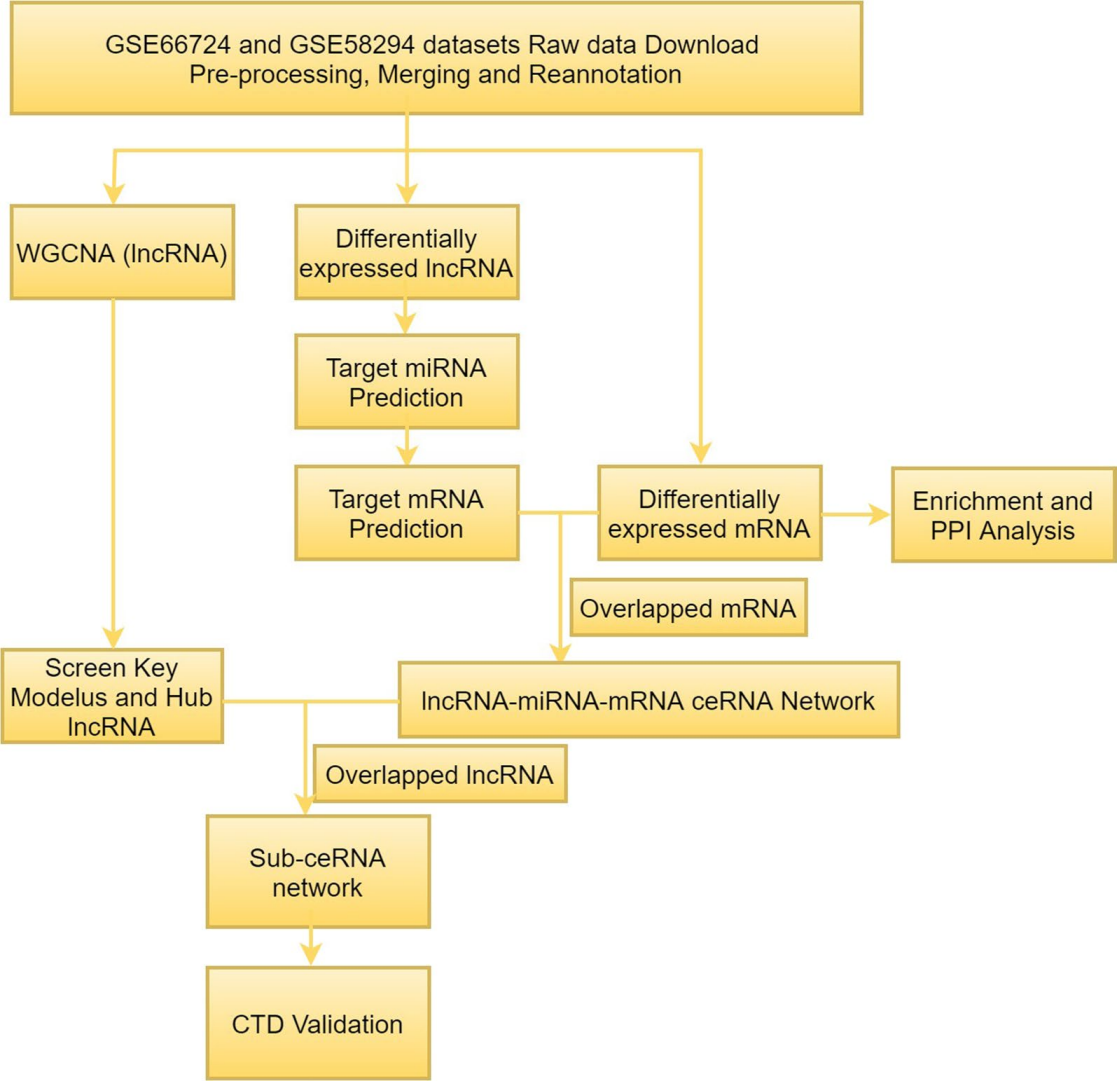
Flowchart of the study. WGCNA, weighted gene co-expression network analysis; PPI, protein-protein interaction; CTD, Comparative Toxicogenomics Database; miRNA, microRNA; lncRNA, long non-coding RNA; ceRNA, competing endogenous RNA

In the current study, we integrated two datasets from the Gene Expression Omnibus (GEO) database. To uncover lncRNAs involved in AFST pathogenesis, it was imperative to combine diverse methods or biology algorithms, thus we conducted a series of analyses including differential expression analysis, Gene Ontology (GO) and Kyoto Encyclopedia of Genes and Genomes (KEGG) pathway enrichment analyses, protein–protein interaction (PPI) network of the differentially expressed mRNA (DEMs) and cluster analysis, WGCNA, ceRNA network analysis, Comparative Toxicogenomics Database (CTD) validation, prognostic analysis based on Receiver operating characteristics (ROC). The workflow was illustrated in Fig. 1.

### Data sources

GEO is a public genomic data repository containing array-based data [21]. Following screening, two datasets of GSE66724 [22] and GSE58294 [23], both of which were annotated using GPL570 [HG-U133_Plus_2] Affymetrix Human Genome U133 Plus 2.0 Array, were downloaded from GEO database. Since the two datasets shared the same platform, these two candidates were selected for the integrated analysis. In all, 16 blood samples were collected from 8 patients with AF but no stroke (AF group), and 8 patients with both AF and stroke (AFST group) in GSE66724. Blood samples of GSE58294 were collected from patients with AF and stroke (AFST group, n = 69) and patients with AF but no stroke (AF group, n = 23). In GSE58294, all blood samples were obtained during the acute phase of the stroke.

### Data preprocessing and probe reannotation

R packages of “affy” and “limma” were applied to assess GSE66724 and GSE58294 RAW data. The data were preprocessed by Robust Multi-array Average (RMA) procedure, and then the data of these two datasets were integrated for the subsequent analysis. Then, we marked different datasets as different batches, and used the “Combat” function in the “sva” package of R software to adjust the batch effect between the two datasets, then the principal component analysis (PCA) cluster plot was drawn to illustrate the samples before and after the batch effect removal. Reannotation of Affymetrix microarray probes to lncRNAs was performed according to the literature [24]. Only lncRNAs with mean expression values > 0.5 in each sample were selected, finally, 1347 lncRNAs were obtained. Before proceeding to the next step, the expression value was normalized using “normalizeBetweenArrays” function in the “limma” package. The repeatability of the data was also validated by the PCA [25]. The PCA and PCA cluster plots were carried out by the “FactoMineR” and “Factoextra” packages.

### Differentially expressed lncRNA (DELs) and differentially expressed mRNAs (DEMs) analyses

The “limma” package was used to explore DELs and DEMs between AFST and AF samples using the empirical bayes method [26]. Benjamin multiple test calibration was used to calculate the false discovery rate (FDR). Finally, the FDR < 0.05 and Fold change (FC) > 1.5 was taken as the threshold to select DELs and DEMs. Thereafter, a volcano plot of the DELs and DEMs was plotted using the “ggplot2” package. A hierarchical cluster heatmap was plotted to represent DEL and DEM expression intensity using the “pheatmap” package.

### Functional enrichment analysis of the DEMs

With GO enrichment analysis, genes could be annotated using dynamic, controlled terms, which were distributed into biological processes (BP), cellular components (CC), and molecular functions (MF). In KEGG analysis, genomic information was linked to higher-order functional information and specific pathways. We used the “clusterProfiler” package to analyze the enrichment of GO terms and KEGG pathways in DEMs. Adjusted *p* value < 0.05 as well as q value < 0.05 were applied as the detection threshold, and the enrichment results were displayed using a dot graph and GOcircle plot.

At the same time, GO enrichment analysis and KEGG enrichment analysis were also performed based on Metascape [27]. The *p*-value < 0.01 was applied as the detection threshold. Then, a network representing the enriched GO terms and KEGG pathways was constructed. The network was visualized using Cytoscape software (V3.6.0) and nodes that represent the enriched terms and pathways were colored according to cluster ID and *p*-value [27]. Based on the DEMs identified in our study, we performed the gene-pathway crosstalk analysis to investigate the interactions among significantly enriched genes and pathways using the ClueGO and Cluepedia plug-in of Cytoscape, and the enriched genes and pathways were mapped into a crosstalk network.

### Identification of protein-protein interaction (PPI) networks of DEMs

PPI network analysis of DEMs was performed using Metascape. A network was constructed when proteins interacted with each other. Subsequently, Cytoscape software (V3.6.0) was applied to visualize and analyze the network, and the topological features including the degree, closeness, betweenness of the nodes in the PPI network were calculated using the CentiScaPe plug-in of Cytoscape. In order to search clusters, the Molecular Complex Detection (MCODE) plug-in of Cytoscape was used.

### CeRNA network construction

The MiRcode database (http://www.mircode.org/), which included presumed interactions between lncRNAs and miRNAs, was used to predict DELs’ relevant target miRNAs [28]. Then, according to the miRTarBase (http://miRTarBase.cuhk.edu.cn/), miRDB (http://mirdb.org), and TargetScan (http://www.targetscan.org) databases [29–31], the aforementioned miRNAs’ relevant target mRNAs were predicted. Only the mRNAs that were identified in all three databases were screened as target mRNAs. In summary, the final ceRNA network contained the DELs, the predicted miRNAs, and the intersection of the target mRNAs and DEMs.

### Identification of Hub lncRNAs through WGCNA

To explore the association between genes and clinical traits, the lncRNA expression matrix was extracted from the merged dataset. All 1347 lncRNAs were chosen to construct the co-expression modules following the instruction of “WGCNA” package [16]. First, we used the “picksoftthreshold” function in the “WGCNA” package to calculate the soft threshold power β for each module. Following the β being settled down, the adjacency matrix was constructed and transformed into a topological overlap matrix (TOM). Then, hierarchical clustering and dynamic tree cut were performed with a merging cut-off value of 0.25 to determine co-expression modules.

The module eigengene (ME) was a weighted average gene expression value and indicated the overall expression level of the module. Then, pearson's correlation analysis was performed on MEs and clinical traits, allowing the identification of the modules which were significantly associated with the external traits. To further verify the module-trait correlation, we also calculated the module significance (MS, defined as the average absolute GS of all genes in the module). In general, modules with high MS values were considered as key modules. For each module, gene significance (GS) represented the association between genes and clinical traits, and module membership (MM) represented the association between genes and MEs. In the key modules, lncRNAs with |GS|> 0.6 and |MM|> 0.5 were identified as AFST related hub lncRNAs.

Using a Venn diagram, the intersection between the hub lncRNAs identified by WGCNA and ceRNA network analysis was determined. Next, using Cytoscape software (V3.6.0), we built a sub-ceRNA regulatory network including the overlapped hub lncRNAs, its target miRNAs, and the downstream mRNAs.

### Further validation of the lncRNAs and mRNAs in the sub-ceRNA network

The CTD (http://ctd.mdibl.org) provided information about the associations between gene products, phenotypes, and diseases [32]. Using the CTD, we were able to identify the potential relationship between lncRNAs and mRNAs in our sub-ceRNA network and the diseases of AF and stroke, with the inference score indicating the strength of association. The genes with high inference scores were identified as having potential clinical implications. Then the expression profiles of the genes were shown and ROC curves were generated to evaluate their diagnostic accuracy, and sensitivity and specificity were assessed using the area under the curve (AUC).

## Results

### Identification of DELs and DEMs in AFST

**Fig. 2:**
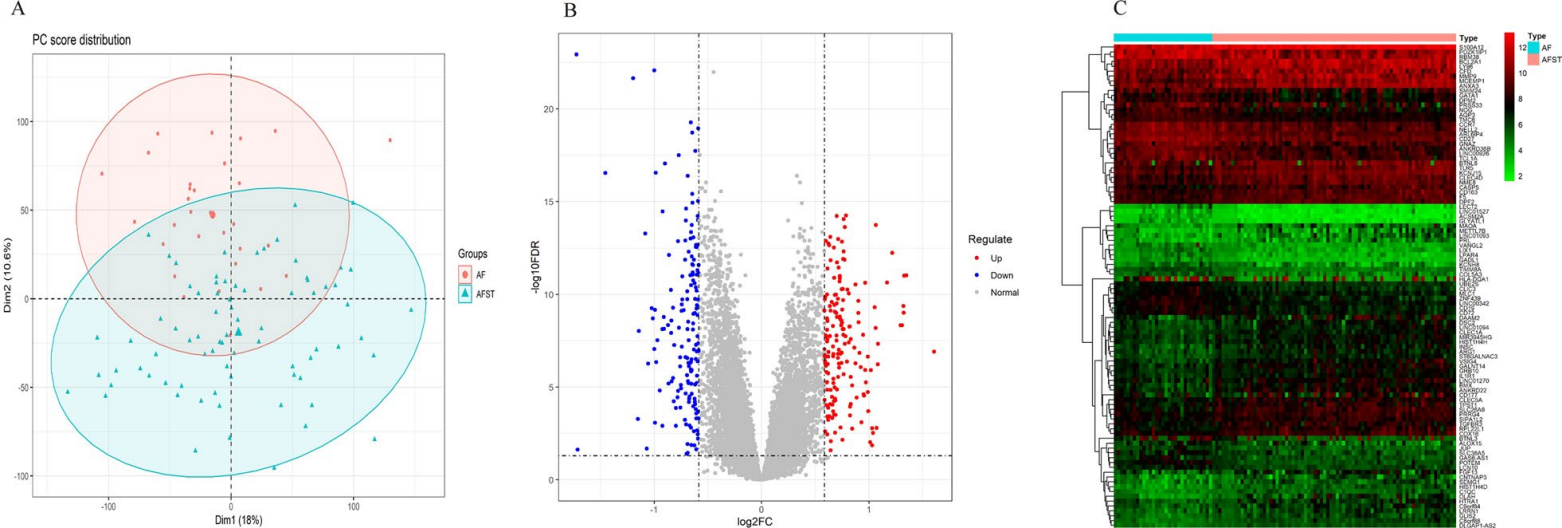
Identification DEMs and DELs in the merged dataset. **A** Principal component analysis plot for the merged dataset. **B** The volcano plot shows the upregulated and downregulated DEMs and DELs in AFST samples. The upregulated DEMs and DELs are highlighted in red, while the downregulated ones are highlighted in blue. The vertical lines represent the |FC| equals to 1.5; and the horizontal line represents the FDR equals to 0.05. **C** Heatmap of the top 100 DELs and DEMs. AF, atrial fibrillation; AFST, atrial fibrillation related stroke; FDR, false discovery rate; FC, fold change; DEMs, differentially expressed mRNAs; DELs, differentially expressed lncRNAs

After data preprocessing, merging, and reannotation of GSE66724 and GSE58294 (Additional files 1 and 2), 54,674 probes corresponding to 18,084 genes, which contained 1347 lncRNAs and 16,737 protein-coding genes, were obtained. According to PCA, significant differences between AF and AFST samples were found (Fig. 2A). Using a threshold of FC > 1.5 and FDR < 0.05, a total of 19 DELs and 317 DEMs were identified between AFST samples and AF samples (Additional files 3 and 4). In the AFST samples, 6 DELs were upregulated, 13 were downregulated; while out of 317 DEMs, 168 were upregulated, 149 were downregulated. A volcano plot and a heatmap of the DELs or DEMs were shown in Fig. 2. In the heatmap, the top 100 DELs or DEMs according to the value of |logFC| were shown and the AFST samples and AF samples were clearly distinguishable from the heatmap.

### Functional enrichment analysis of the DEMs

**Fig. 3:**
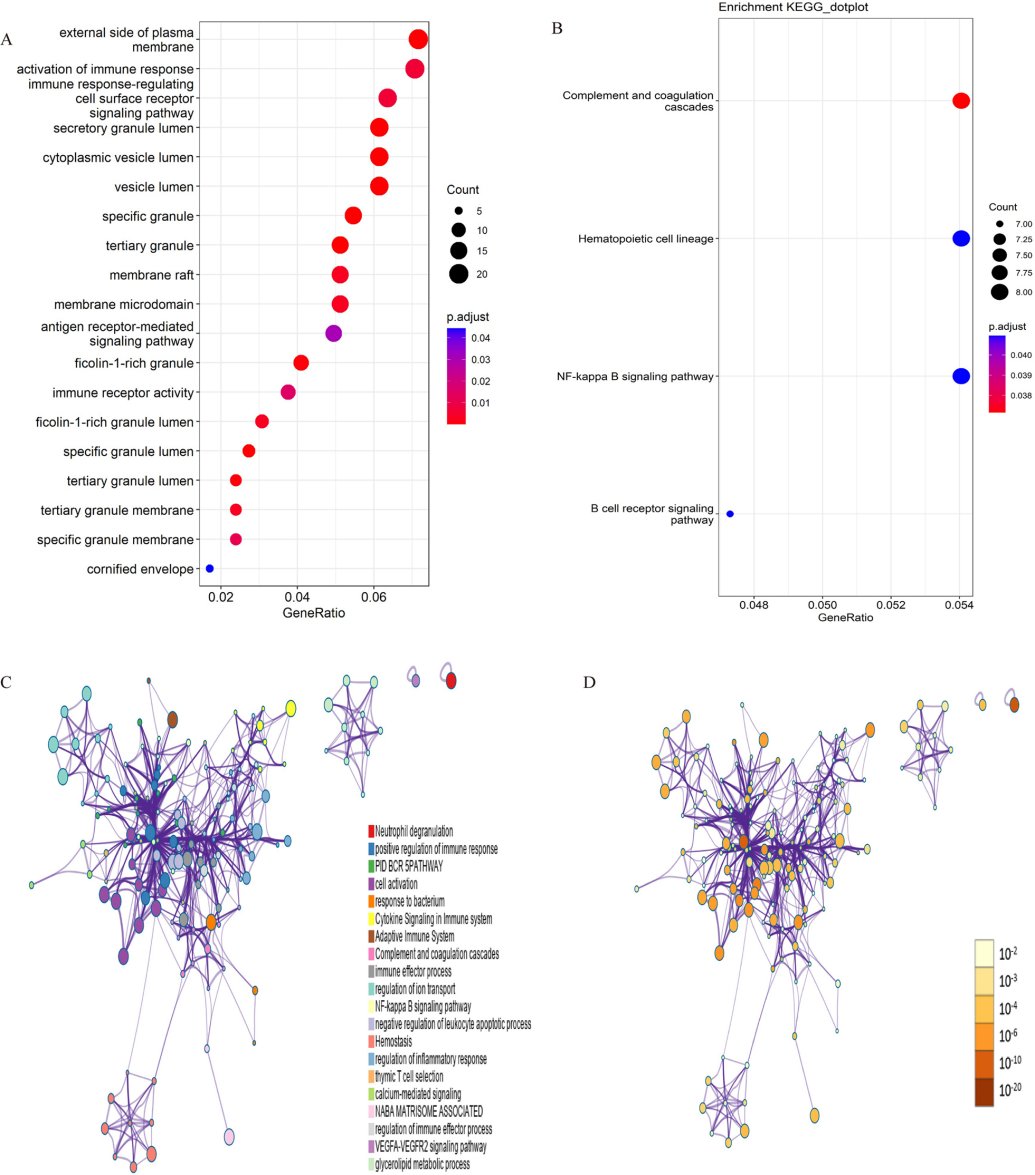
The functional enrichment analysis of the DEMs. **A** GO enrichment analysis. **B** KEGG pathway enrichment analysis. In **A** and **B**, the dot color reflects the level of significance, whereas the dot size reflects the number of target genes enriched in the corresponding pathway. **C** Network of enriched terms analyzed by Metascape (colored by cluster ID). **D** Network of enriched terms analyzed by Metascape (colored by p-value). In **C** nodes share the same cluster ID are typically close to each other. In **D**, the deeper of the color, the more significant of the p-value. GO, Gene Ontology; KEGG, Kyoto Encyclopedia of Genes and Genomes; DEMs, differentially expressed mRNAs

The enrichment analyses of GO and KEGG pathways with a cut-off value of adjusted *p*-value < 0.05 as well as q-value < 0.05 were presented in Additional files 5 and 6, where the top 20 GO terms and KEGG pathways were shown according to the adjusted p-value. As shown in Fig. 3A and Additional file 7, activation of immune response, immune response-regulating cell surface receptor signaling pathway, and antigen receptor-mediated signaling pathway were dominant enriched BP terms, meanwhile, the enriched MF term was immune receptor activity. The concentric circle diagram of the GO analysis was shown in Additional file 8. Moreover, the KEGG enrichment analysis showed that the complement and coagulation cascade was the most enriched pathway, followed by hematopoietic cell lineage, NF-kappa B signaling pathway, B cell receptor signaling pathway (Fig. 3B). The significantly enriched terms and pathways might contribute to a further understanding of the role played by DEMs in AFST.

**Fig. 4:**
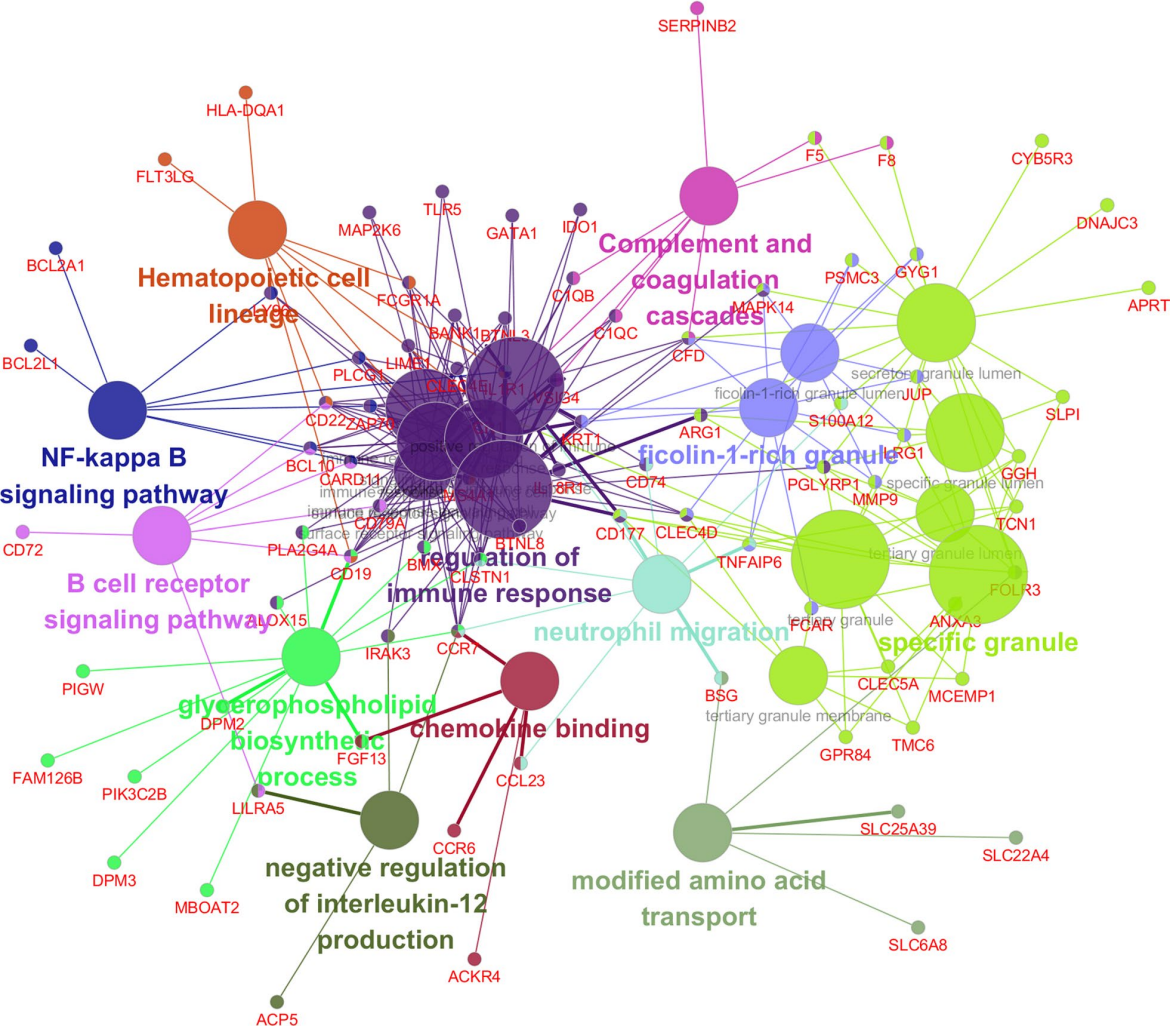
Gene-pathway crosstalk network. The large circles represent pathways, and the size of large circles indicates the level of significance of the pathway, and the pathways are grouped according to the kappa score. The small circles represent genes, and the thickness of the lines indicates the strength of the interaction

Additionally, we used Metascape to analyze functional enrichment, and the enriched terms were integrated into the networks by cluster ID and p-value. Nodes with the same cluster ID were colored the same in Fig. 3C, and terms enriched with more genes tended to be more significant in Fig. 3D. At the same time, we performed the gene-pathway crosstalk analysis to investigate the interactions among significantly enriched genes and pathways using the ClueGO and Cluepedia plug-in of Cytoscape, a gene-pathway network was constructed to visualize the associations between the significantly enriched pathways and genes (Fig. 4).

As a result of the enrichment analysis described above, the DEMs associated with AFST were mainly enriched in the activation of immune response and complement and coagulation cascades. The results showed that AFST might be closely associated with the process of immune response and complement and coagulation cascades.

### PPI network and cluster analysis

In order to better understand the DEM interactions, we used Metascape to analyze PPI network. The PPI network was composed of 216 nodes and 339 edges (Additional file 9), and the topological features including the degree, closeness, and betweenness of the nodes in the PPI network were showed in Additional file 10. Then we used the MCODE plug-in of Cytoscape to search for clusters in the network. Finally, according to k-core = 2, four clusters were identified (Additional files 9 and 11).

### Construction of the ceRNA network

First, the miRcode database was applied to predict miRNAs interacting with DELs. In all, 165 interactions between 4 DELs and 109 unique miRNAs were determined (Additional file 12). Following that, the target mRNAs of the 109 miRNAs were predicted using the miRTarBase, miRDB, and TargetScan databases. In total, 688 interactions between 109 miRNAs and 599 distinct mRNAs were identified (Additional file 13). Based on the overlapped mRNAs of the 599 mRNAs and 317 DEMs, a ceRNA network consisting of 3 lncRNAs, 7 miRNAs, and 11 mRNAs was constructed (Table 1, Additional file 14). All the three lncRNAs (*LINC00323, LINC00342, GAS6-AS1*) were downregulated in AFST patients.

**Table 1:** CeRNA network of lncRNAs, miRNAs and mRNAs in AFST

lncRNAs	miRNAs	mRNAs
*LINC00323*	*hsa-miR-507*	*BCL7A*
	*hsa-miR-363-3p*	*GOLGA8A, SERTAD3, LHFPL2*
	*hsa-miR-107*	*TGFBR3*
	*hsa-miR-33a-3p*	*DLGAP5*
*LINC00342*	*hsa-miR-142-3p*	*SLC37A3, C9orf72*
	*hsa-miR-27a-3p*	*TGFBR3, ABCA1*
	*hsa-miR-129-5p*	*EBF1*
*GAS6-AS1*	*hsa-miR-363-3p*	*GOLGA8A, SERTAD3, LHFPL2*
	*hsa-miR-507*	*BACH2, BCL7A*

*ceRNA* Competing endogenous RNA, *AFST* Atrial fibrillation related stroke, *lncRNA* Long non-coding RNA, *miRNA* microRNA, *mRNA* messenger RNA

### Identification hub lncRNAs through WGCNA

**Fig. 5:**
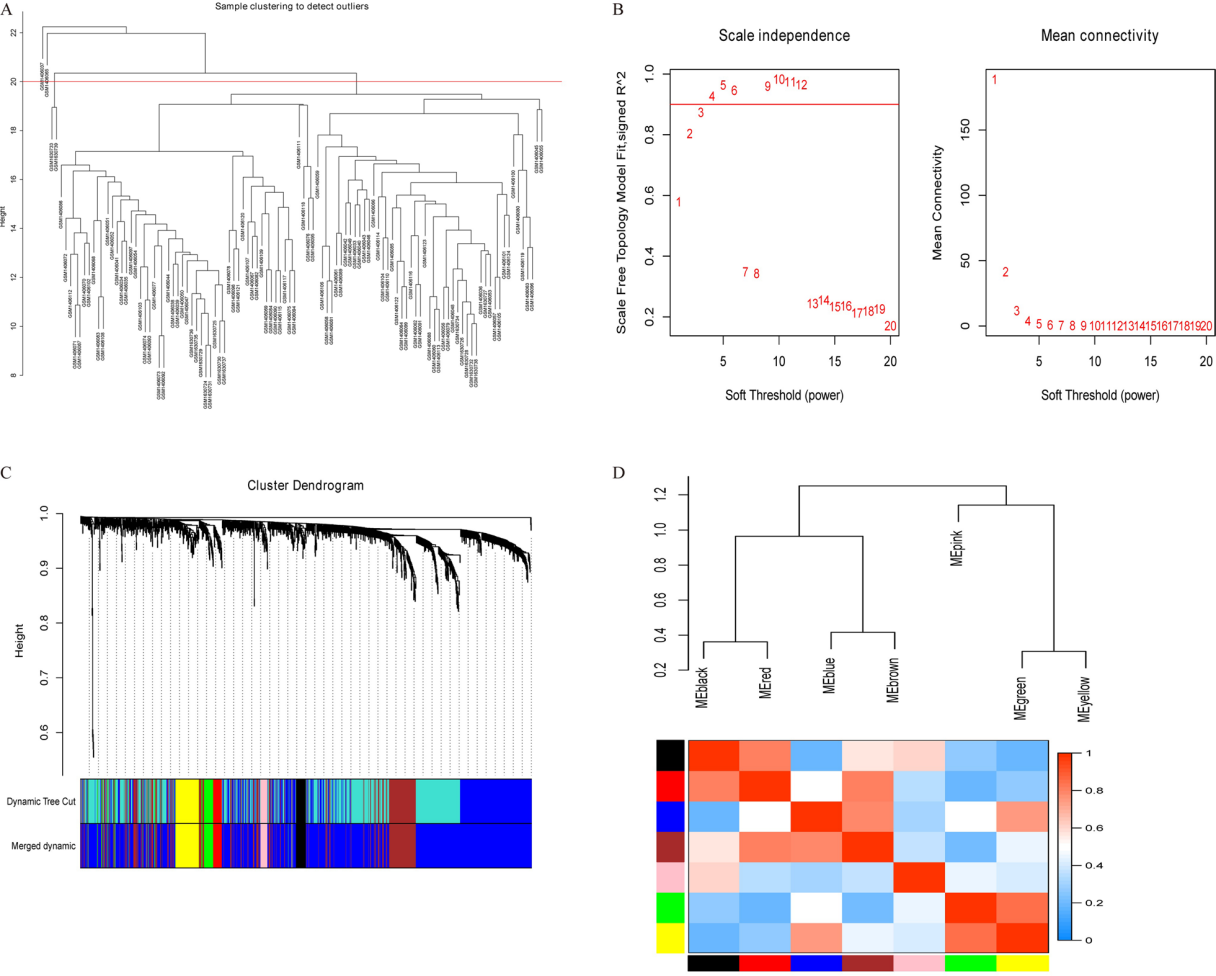
Construction of Co-expression modules used WGCNA. **A** Sample clustering to detect outliers. The red line represents the threshold for outlier. **B** Soft-threshold power analysis. The left picture shows the scale free fit index for each soft-thresholding power. The right picture displays the mean connectivity for each soft-thresholding power. **C** Co-expression cluster dendrogram, based on TOM similarity. Each color represents one module. **D** Module eigengene clustering and eigengene adjacency heatmap, which shows the correlation between each module. TOM; topological overlap matrix; WGCNA, weighted gene co-expression network analysis

In order to further verify the hub lncRNAs, we performed WGCNA in which all 1347 lncRNAs were included to construct the co-expression modules. The samples were analyzed using hierarchical clustering, and four obvious outliers (GSM1406037, GSM1406065, GSM1630733, GSM1630739) were removed from the cohort before WGCNA (Fig. 5A). It was shown in Fig. 5B that a threshold power of 3 was sufficient for WGCNA. As illustrated in Fig. 5C, the final 7 modules were identified based on a hierarchical clustering and dynamic tree cutting algorithm (cut-off value was 0.25). The largest module (blue) contained 906 lncRNAs while the smallest one (pink) contained 21 lncRNAs. By WGCNA, genes without a distinct module assignment were grouped in a gray module and were dismissed in the following analysis. Furthermore, interactions between the seven modules were analyzed. Together with the eigengene adjacency heatmap, the dendrogram of the modules demonstrated a high level of co-expression module independence (Fig. 5D).

**Fig. 6:**
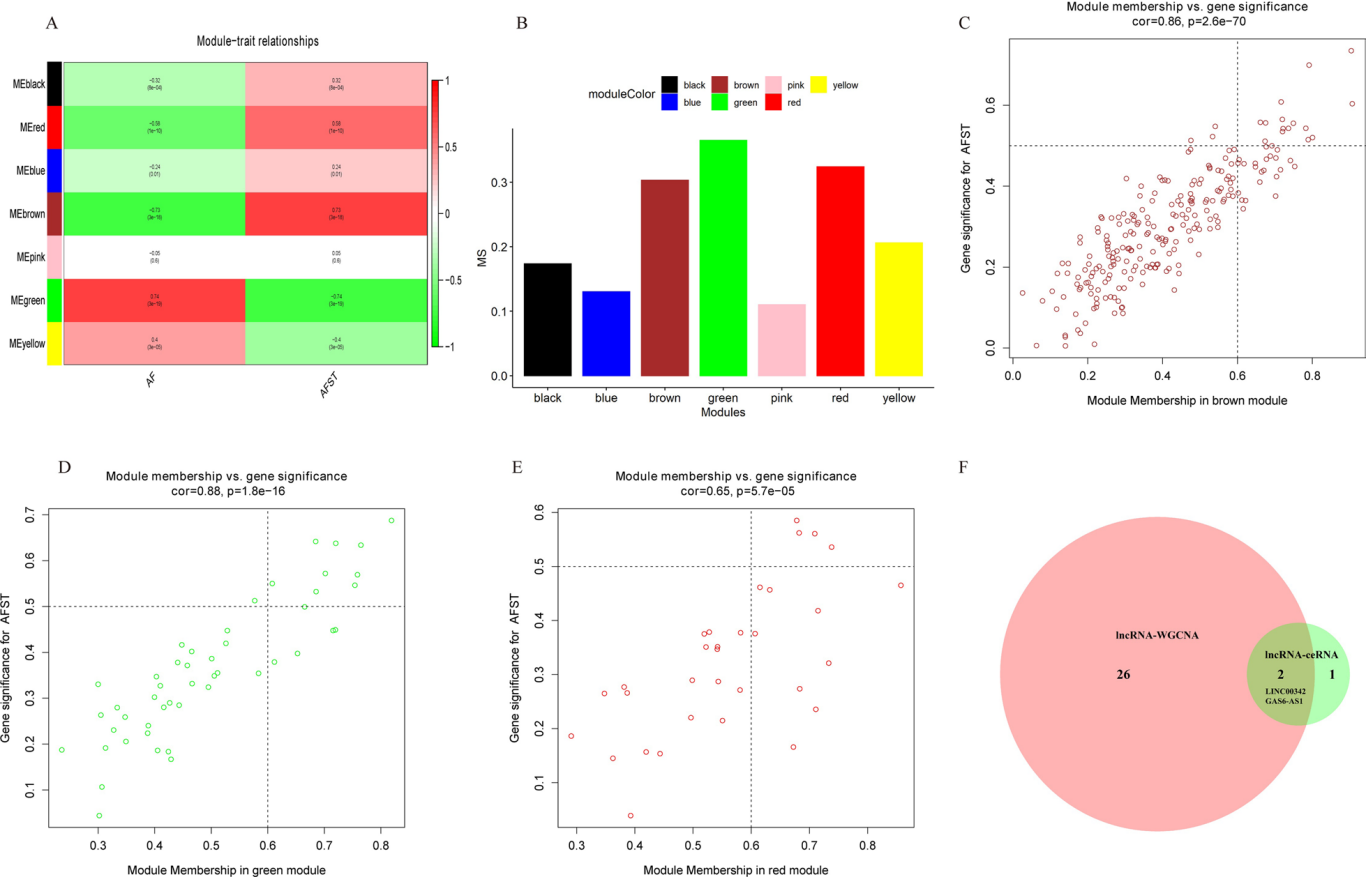
Identification of AFST related module and hub lncRNAs by WGCNA. **A** Heatmap of the correlation between the MEs and clinic traits. The Green module and the brown module are the most relevant modules with AFST. **B** Barplot of the MS across modules related to AFST. **C** Scatter plot between GS for AFST and the MM in brown module. **D** Scatter plot between GS for AFST and the MM in green module. **E** Scatter plot between GS for AFST and the MM in red module. **F** A Venn diagram of the lncRNAs identified in ceRNA network analysis and WGCNA. The overlap between lncRNAs in ceRNA network and lncRNAs with |GS|> 0.6 and |MM|> 0.5 in brown, green and red modules represent the hub lncRNAs for further validation. lncRNA, long non-coding RNA; AFST, atrial fibrillation related stroke; ME, module eigengene; MS, module significance; GS, gene significance; MM, module membership; ceRNA, competing endogenous RNA; WGCNA, weighted gene co-expression network analysis

Using correlation analysis, we investigated the relationship between modules and external traits. The green module had the most negative correlation with AFST (r = − 0.74), while the brown module had the most positive correlation with AFST (r = 0.73). (Fig. 6A). Moreover, across all modules, the green module had the highest MS values, followed by the red module and the brown module (Fig. 6B). Therefore, taking together the results of correlation analysis and MS, the red module, green module, and brown module were identified as the core modules for AFST. In addition, the genes in the 3 modules were analyzed using GS and MM. The genes in the upper right section of Fig. 6C–E, which had high values of GS and MM, were significantly associated with AFST and were the most important elements of the three modules at the same time. Consequently, a total of 28 lncRNAs (Table 2) in the upper right section of Fig. 6C–E were considered for further analysis.

**Table 2:** Hub lncRNAs identified in WGCNA

lncRNAs	GS	*p*-value (GS)	MM	*p*-value (MM)	Module color
*LINC00926*	− 0.556	0.000	− 0.749	0.000	Brown
*SEPSECS-AS1*	− 0.558	0.000	− 0.661	0.000	Brown
*LINC00342*	− 0.735	0.000	− 0.904	0.000	Brown
*ST20-AS1*	0.542	0.000	0.722	0.000	Brown
*DLGAP1-AS2*	0.699	0.000	0.791	0.000	Brown
*FAM13A-AS1*	0.557	0.000	0.675	0.000	Brown
*ZNF790-AS1*	− 0.535	0.000	− 0.720	0.000	Brown
*TSPOAP1-AS1*	− 0.604	0.000	− 0.907	0.000	Brown
*DANCR*	− 0.565	0.000	− 0.720	0.000	Brown
*EPB41L4A-AS1*	− 0.543	0.000	− 0.783	0.000	Brown
*ZFAS1*	0.608	0.000	0.717	0.000	Brown
*CKMT2-AS1*	− 0.515	0.000	− 0.789	0.000	Brown
*TNRC6C-AS1*	− 0.543	0.000	− 0.736	0.000	Brown
*TPT1-AS1*	− 0.511	0.000	− 0.674	0.000	Brown
*HCG18*	− 0.520	0.000	− 0.801	0.000	Brown
*GAS6-AS1*	− 0.550	0.000	0.608	0.000	Green
*LINC01527*	− 0.532	0.000	0.685	0.000	Green
*LINC00624*	− 0.642	0.000	0.685	0.000	Green
*DUBR*	− 0.572	0.000	0.702	0.000	Green
*LINC00550*	− 0.688	0.000	0.819	0.000	Green
*LINC00276*	− 0.638	0.000	0.720	0.000	Green
*KRBOX1-AS1*	− 0.546	0.000	0.754	0.000	Green
*C17orf77*	− 0.569	0.000	0.759	0.000	Green
*DSG2-AS1*	− 0.634	0.000	0.765	0.000	Green
*SSBP3-AS1*	− 0.561	0.000	− 0.709	0.000	Red
*LINC01089*	− 0.536	0.000	− 0.738	0.000	Red
*ARRDC1-AS1*	− 0.562	0.000	− 0.682	0.000	Red
*CCDC18-AS1*	− 0.585	0.000	− 0.678	0.000	Red

*WGCNA* Weighted gene co-expression network analysis, *GS* Gene significance, *MM* Module membership, *lncRNA* Long non-coding RNA

**Fig. 7:**
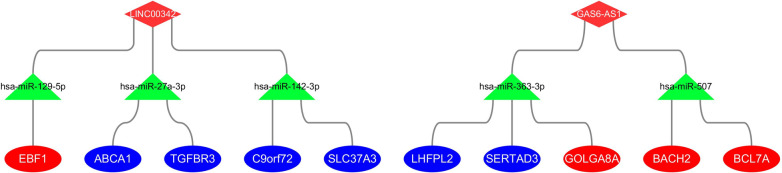
Construction of the AFST-related lncRNA-miRNA-mRNA sub-ceRNA network. Rhombuses represent lncRNAs, triangles represent miRNAs and ellipses represent mRNAs, respectively. Red and blue color represent down-regulation and up-regulation, respectively. According to ceRNA theory, lncRNAs are supposed to regulate mRNAs positively, so only the genes with the same color (red) in the network are in accordance with the theoretical expectation. ceRNA, competing endogenous RNA; AFST, atrial fibrillation related stroke; lncRNA, long non-coding RNA; miRNA, microRNA; mRNA, messenger RNA

The overlapped lncRNAs of the three lncRNAs in the ceRNA network and the 28 lncRNAs identified through WGCNA, *GAS6-AS1* and *LINC00342*, were identified as hub lncRNAs (Fig. 6F). These two lncRNAs, together with their target miRNAs and mRNAs, were applied to construct a sub-ceRNA network (Fig. 7). According to ceRNA theory, as miRNA sponges, lncRNAs were supposed to regulate mRNAs positively. In our sub-ceRNA network, two downregulated lncRNAs (*GAS6-AS1*, *LINC00342*) and four downregulated mRNAs (*BCL7A*, *BACH2*, *GOLGA8A*, *EBF1*) aligned with the ceRNA theory, and were considered for further investigation.

### Further validation of the lncRNAs and mRNAs in the sub-ceRNA network

Then, using the CTD, we predicted the potential role of the aforementioned six genes in AF and stroke. The inference score for the RNAs targeted AF and stroke was shown in Table 3. Finally, one lncRNA, *GAS6-AS1*, and three mRNAs including *BCL7A*, *BACH2*, *GOLGA8A* turned out to be associated with AFST based on ceRNA network analysis and WGCNA, as well as CTD validation. *GAS6-AS1* might function, at least in part, as a ceRNA to regulate *BCL7A*, *BACH2*, and *GOLGA8A* in AFST.

**Table 3:** Inference score between hub genes and AF or stroke

Hub genes	Classification	AF	Stroke
*GAS6-AS1*	lncRNA	6.03	6.79
*LINC00342*	lncRNA	NA	NA
*BCL7A*	mRNA	15.17	21.53
*BACH2*	mRNA	10.56	34.99
*EBF1*	mRNA	9.35	21.07
*GOLGA8A*	mRNA	9.3	26.37

*AF* Atrial fibrillation, *lncRNA* Long non-coding RNA, *mRNA* messenger RNA, *NA* Not available

The expression levels of the four hub genes were shown in Additional file 15, which showed that *GAS6-AS1*, *BCL7A*, *BACH2*, and *GOLGA8A* expression were significantly lower in the AFST samples compared with the AF samples. Subsequently, ROC curves were performed to assess the diagnostic value of the hub genes for AFST, and it was shown that the AUC for *GAS6-AS1* was 0.828. Similar results for *BCL7A*, *BACH2*, and *GOLGA8A* were presented in Additional file 16.

## Discussion

In the current study, 31 blood samples from AF patients and 77 blood samples from AFST patients were enrolled from two datasets. For the first time, we found that lncRNA *GAS6-AS1* might be associated with AFST. Both ceRNA network analysis and WGCNA were performed to confirm the role of *GAS6-AS1* in AFST. The two different methods yielded identical results regarding the function of *GAS6-AS1* in AFST, which was further confirmed by CTD. The reliable results indicated that lncRNA *GAS6-AS1* might be a potential predictor of AFST or a potential therapeutic target in treating AFST.

Several studies had assessed the biomarkers in AFST previously. It was suggested by Allende et al. [22] that Hsp70 protected AFST patients by preventing thrombosis without increasing bleeding risk and it would be a new target to treat AFST patients. Using the datasets of GSE79768 and GSE58294, Zou et al. [33] found that the expression of *ZNF566*, *PDZK1IP1*, *ZFHX3*, and *PITX2* genes were related to AFST and may be potential therapeutic targets for it. Based on the datasets of GSE66724 and GSE58294, Zhang et al. [34] found that ten genes including *SMURF2*, *CDC42*, *UBE3A*, *RBBP6*, *CDC5L*, *NEDD4L*, *UBE2D2*, *UBE2B*, *UBE2I*, and *MAPK1* were overexpressed in AFST patients. According to Li et al. [35], the factor of inflammation was supposed to be considered when treating AFST patients, and certain genes, including *MEF2A*, *CAND1*, *PELI1*, and *PDCD4* were identified and might contribute to the pathogenesis of AFST. The inconsistency of the hub genes in different studies might be attributed to the different samples included and different analysis protocols. It was intriguing that all the aforementioned studies focused on the differentially expressed mRNAs, to our knowledge, no previous study had investigated the role of lncRNA in AFST.

In 1988, for the first time, Schneider and his colleagues identified six members of the growth-arrest-specific (GAS) family of genes [36]. Located on chromosome 13q34, the *GAS6* gene has been shown to contribute to cell proliferation. An antisense RNA of *GAS6*, named *GAS6-AS1*, which is transcribed from chromosome 13q34 too, also plays an important role in the pathogenesis of many kinds of cancers. In different cancers, the role of *GAS6-AS1* on patients' prognosis is extensively inconsistent. *GAS6-AS1* may play a tumor suppressor role in lung cancer [37]. Similarly, a higher level of *GAS6-AS1* expression is associated with a better survival in Non-Small-Cell Lung Cancer (NSCLC) patients [38]. Nevertheless, *GAS6-AS1* promotes the migration and proliferation of gastric cancer cells by enhancing their entry into S-phase [39]. By sponging *miR-370-3p*, *GAS6-AS1* contributes to the development of acute myeloid leukemia [40]. The opposite results that both the oncogenic [41] effect and anti-oncogenic [42] effect are obtained in papillary renal cell carcinoma.

The role of *GAS6-AS1* in stroke has rarely been investigated. It’s suggested that *GAS6-AS1* may be related to an increased risk of HT after intravenous thrombolysis in acute ischemic stroke patients [43]. In the current study, the association between *GAS6-AS1* and AFST is reported for the first time. *GOLGA8A*, one of the target mRNAs of *GAS6-AS1* in our ceRNA network, has been shown to be related to intracerebral hemorrhage too [44]. So, the *GAS6-AS1*/*hsa-miR-363-3p*/*GOLGA8A* axis in our ceRNA network seems to be related to intracerebral hemorrhage. Meanwhile, AFST is characterized by a high percentage of HT in the days immediately after the stroke [7]. Therefore, it is plausible to postulate an association between the *GAS6-AS1/hsa-miR-363-3p/GOLGA8A* axis and HT after AFST, which warrants further investigation.

Increasing evidence suggests that ischemic stroke is associated with profound immune responses in the blood and the activation of multiple immune cell subsets. However, there is still a debate over whether these immune responses are beneficial or detrimental [45]. Therefore, it is crucial to identify specific molecular targets to develop a new immunomodulatory treatment to prevent the detrimental effect of immune responses after stroke [46]. Functional enrichment analyses in our study reveal that the DEMs related to AFST are primarily enriched in the biological processes of activation of the immune response and complement and coagulation cascades. The result proposes that AFST may be correlated with the process of immune response. Therefore, the hub genes identified in our study may be the molecular targets that we are looking for to develop new immunomodulatory therapies.

Among the three target mRNAs of *GAS6-AS1* in our ceRNA network, *BACH2* has higher inference scores for AF and Stroke, at the same time, Bach2 has been suggested as an influential immune-regulating transcription factor in T helper 2 (Th2), Follicular T helper (Tfh), regulatory T cell (Treg), B cells and plays a key role in Th2 immune response previously [47]. *BCL7A* tends to be related to cancer [48], but not stroke. Taking into account the inference scores and the biological function of the target mRNAs, it is possible that *GAS6-AS1* downregulation may function in AFST patients by regulating *BACH2* as a ceRNA through the immune response.

Collectively, we thus propose an association between the ceRNA axis *GAS6-AS1/hsa-miR-363-3p/GOLGA8A* and HT after AFST, and predict that the *GAS6-AS1/hsa-miR-507/BACH2* axis has a potential role in AFST through inflammatory and immune responses. They may be potential targets for AFST therapy. The detailed mechanisms may need further investigation.

There are still limitations in our current study. First, although different approaches have been used to demonstrate the role of lncRNA *GAS6-AS1*, further validation is needed to confirm it. Second, due to the lower expression levels of lncRNAs compared to mRNAs, WGCNA is performed only for lncRNAs, and as a result, lncRNA-mRNA interactions may be missing. Most importantly, the potential mechanisms of the association between GAS6-AS1 and AFST was speculated on the basis of previous studies and bioinformatics analysis. Further experiments (both in vivo and in vitro) are desperately needed to verify our findings. In addition, gene expression differs in different stroke phase [49]. All blood samples in GSE58294 are taken during the acute phase of the stroke, and we cannot rule out that samples taken at a different stroke phase may have yielded different results.

## Conclusions

In conclusion, we identified a hub lncRNA of *GAS6-AS1* associated with AFST by ceRNA network analysis and WGCNA. It was subsequently validated by CTD that *GAS6-AS1* played a pivotal role in AFST. These findings suggested that low expression of *GAS6-AS1* might exert an essential role in AFST through downregulating *GOLGA8A* and *BACH2*, by affecting post-AFST hemorrhagic transformation and post-AFST immune response, and pointed out the direction for further research. Altogether, these analyses suggested that *GAS6-AS1* might represent a potential target for AFST therapy.

## Supplementary Information

**Additional file 1**.** FigS1**. Data distribution. (A) Data distribution of GSE66724 before normalization (B) Data distribution of GSE58294 before normalization (C) Data distribution of the merged dataset after data normalization.

**Additional file 2. FigS2**. PCA plot of the data before and after the batch effect removal. (A) PCA results before the batch effect removal. (B) PCA results after the batch effect removal.

**Additional file 3**. Differentially expressed lncRNAs.

**Additional file 4**. Differentially expressed mRNAs.

**Additional file 5**. GO enrichment analysis of the DEMs.

**Additional file 6**. KEGG enrichment analysis of the DEMs.

**Additional file 7**.** FigS3**. GO terms plot of the DEMs, Colors in different plots indicate the level of significance. (A) Biological processes (B) Cellular components (C) Molecular functions.

**Additional file 8**.** FigS4**. Circle plot of the GO enrichment analysis. The left outer semicircle represents the logFC value of the genes, and the right semicircle corresponds to GO terms enriched.

**Additional file 9**.** FigS5**. Clusters of the PPI network based on the Metascape and MCODE analysis. Four colors of red, bule, yellow and green indicate four clusters identified by MCODE analysis.

**Additional file 10**. Topological features of the nodes in the PPI network.

**Additional file 11**. Genes in different cluster identified by MCODE.

**Additional file 12**. lncRNA target miRNA prediction.

**Additional file 13**. miRNA target mRNA prediction.

**Additional file 14**.** FigS6**. CeRNA regulatory network. Red rhombuses represent lncRNAs, green triangles represent miRNAs and blue circles represent mRNAs, respectively.

**Additional file 15**.** FigS7**. Boxplot of the expression level for four hub genes.

**Additional file 16**.** FigS8**. The receiver operator characteristic curves of GAS6-AS1, GOLGA8A, BACH2 and BCL7A for AFST.

## Data Availability

The datasets generated for this study can be found in the GEO database (GSE66724 and GSE58294; https://www.ncbi.nlm.nih.gov/geo/).

## Abbreviations

AF: Atrial fibrillation
AFST: Atrial fibrillation related stroke
AUC: The area under the curve
*BACH2*: BTB domain and CNC homolog 2
*BCL7A*: B-cell CLL/lymphoma 7 protein family, member A
BP: Biological processes
CC: Cellular components
ceRNA: Competing endogenous RNA
COPD: Chronic obstructive pulmonary disease
CTD: Comparative toxicogenomics database
DEMs: Differentially expressed mRNAs
DELs: Differentially expressed lncRNAs
FC: Fold change
FDR: False discovery rate
*GAS6-AS1*: Growth-arrest-specific 6-antisense RNA1
GEO: Gene expression omnibus
GO: Gene ontology
GOLGA8A: Golgin A8 family, member A
GS: Gene significance
HT: Hemorrhagic transformation
KEGG: Kyoto encyclopedia of genes and genomes
lncRNA: Long non-coding RNA
MCODE: Molecular complex detection
ME: Module eigengene
MF: Molecular functions
miRNA: MicroRNA
MM: Module membership
mRNA: Messenger RNA
MS: Module significance
NSCLC: Non-small-cell lung cancer
PCA: Principal component analysis
PPI: Protein-protein interaction
RMA: Robust multi-array average
ROC: Receiver operating characteristic
Tfh: Follicular T helper
Th2: T helper 2
TOM: Topological overlap matrix
Treg: Regulatory T cell
WGCNA: Weighted gene co-expression network analysis
