# Supplementary material for: GAS6-AS1, a long noncoding RNA, functions as a key candidate gene in atrial fibrillation related stroke determined by ceRNA network analysis and WGCNA
Source: BMC Med Genomics. 2023 Mar 9;16:51. doi: 10.1186/s12920-023-01478-y (PMC9996875; doi:10.1186/s12920-023-01478-y)
Supplement: Supplementary file 1 — Additional file 1. FigS1. Data distribution. (A) Data distribution of GSE66724 before normalization (B) Data distribution of GSE58294 before normalization (C) Data distribution of the merged dataset after data normalization. [file 12920_2023_1478_MOESM1_ESM.zip › Additional file 1 legend.docx]

Additional file 1: FigS1 Data distribution. (A) Data distribution of GSE66724 before normalization (B) Data distribution of GSE58294 before normalization (C) Data distribution of the merged dataset after data normalization.
