# Supplementary figures and images for: GAS6-AS1, a long noncoding RNA, functions as a key candidate gene in atrial fibrillation related stroke determined by ceRNA network analysis and WGCNA
Source: BMC Med Genomics. 2023 Mar 9;16:51. doi: 10.1186/s12920-023-01478-y (PMC9996875; doi:10.1186/s12920-023-01478-y)

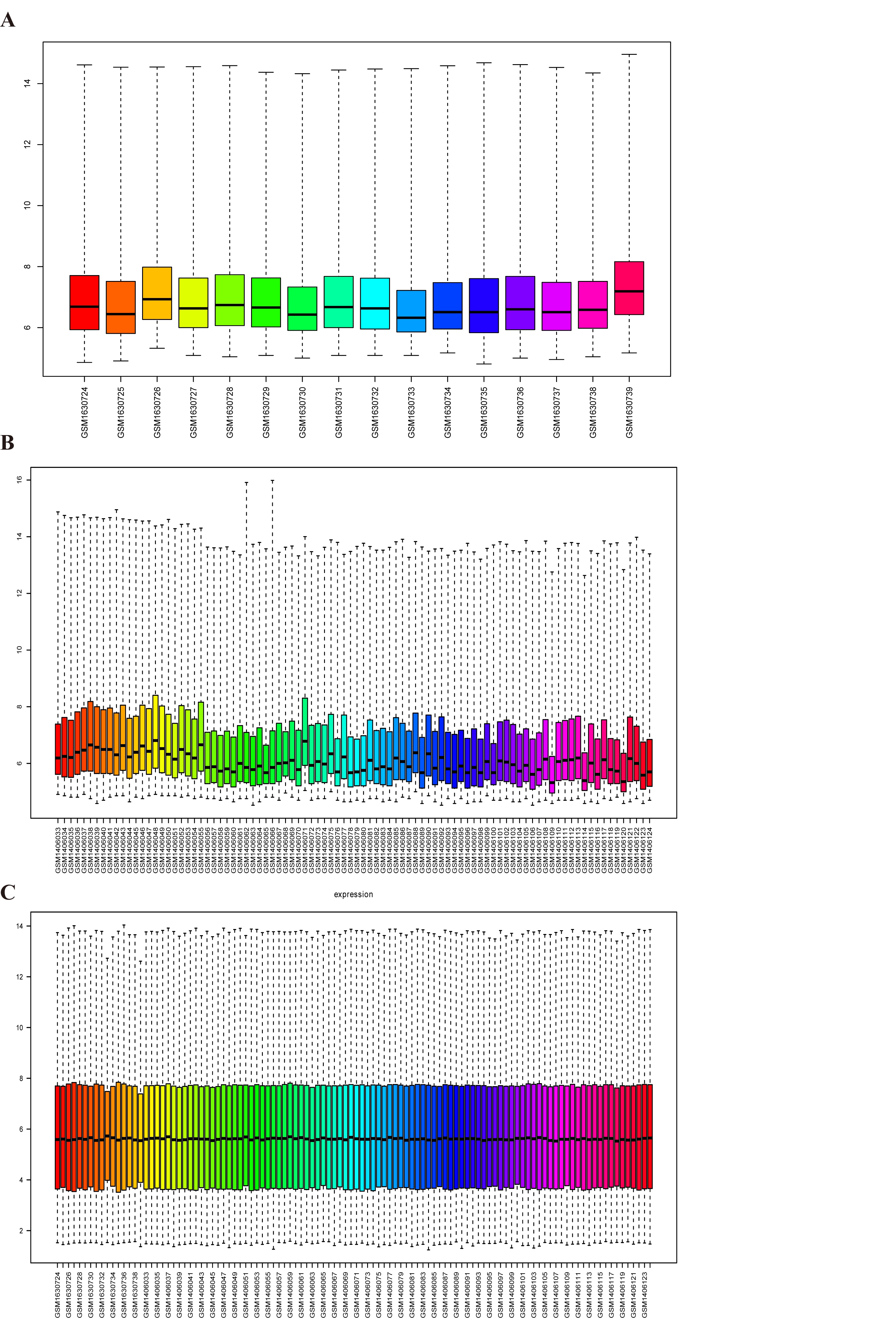

Supplement: Supplementary file 1 — Additional file 1. FigS1. Data distribution. (A) Data distribution of GSE66724 before normalization (B) Data distribution of GSE58294 before normalization (C) Data distribution of the merged dataset after data normalization. [file 12920_2023_1478_MOESM1_ESM.zip › Additional file 1.jpg]

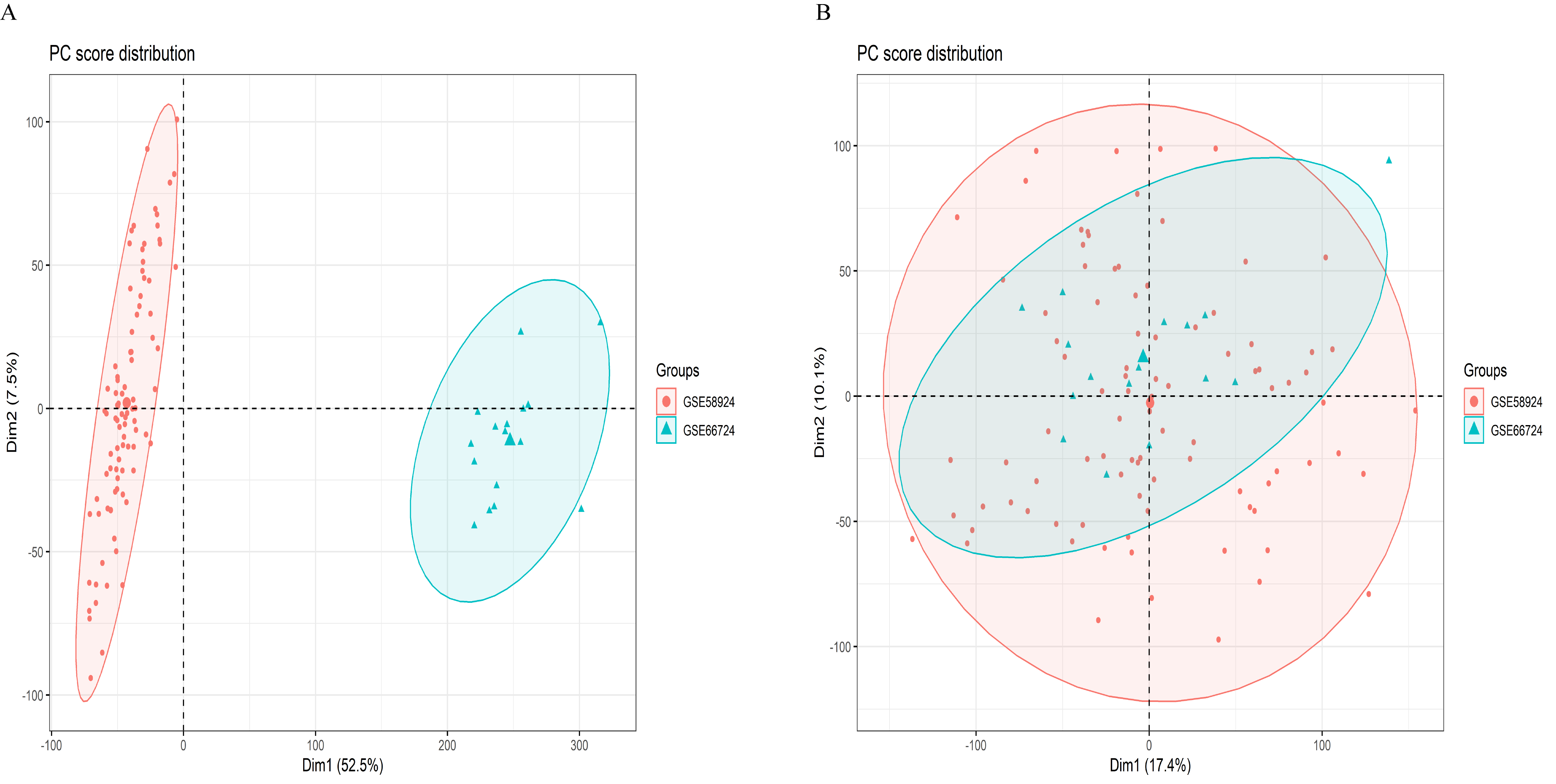

Supplement: Supplementary file 2 — Additional file 2. FigS2. PCA plot of the data before and after the batch effect removal. (A) PCA results before the batch effect removal. (B) PCA results after the batch effect removal. [file 12920_2023_1478_MOESM2_ESM.zip › Additional file 2.tif]

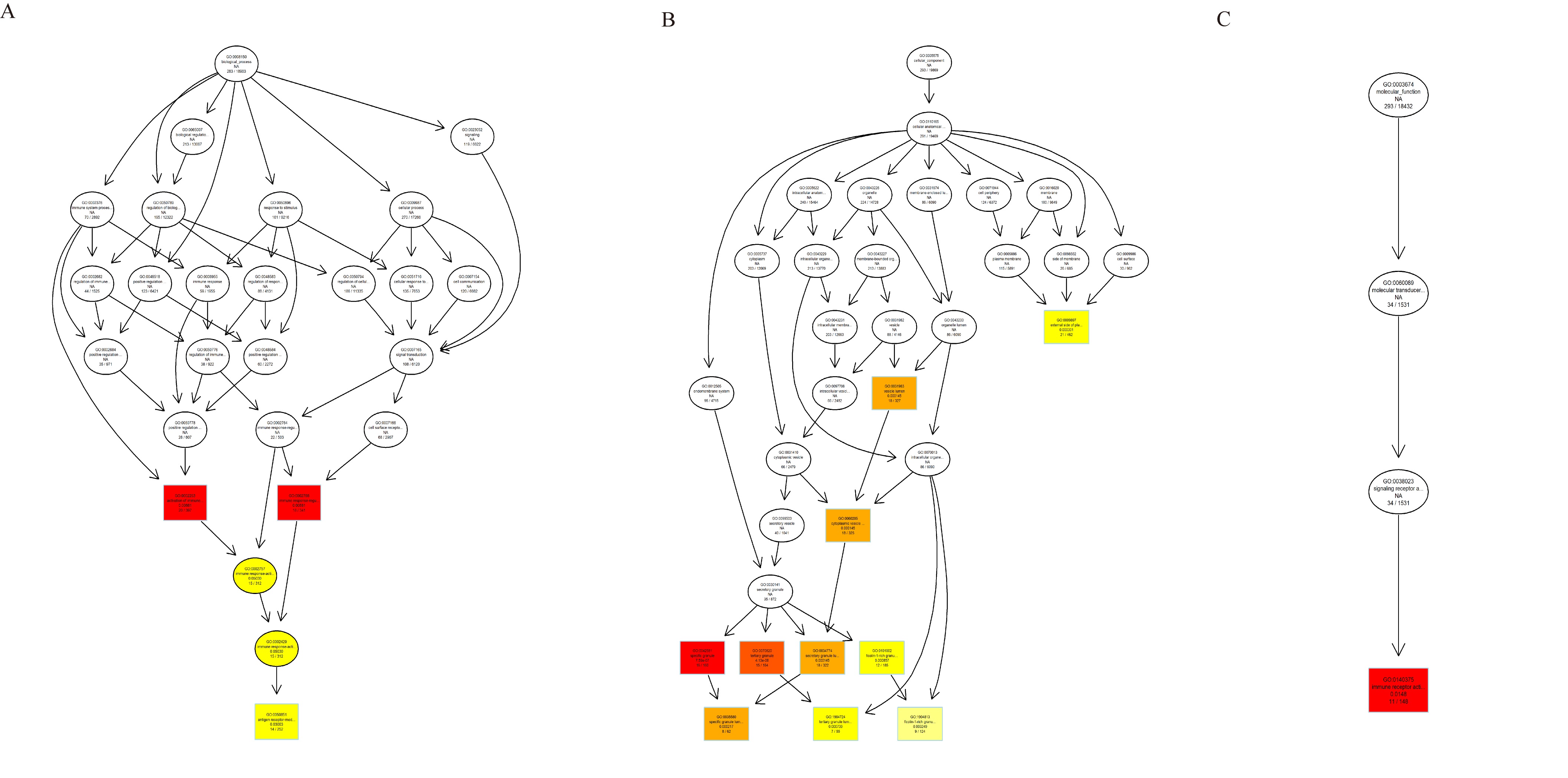

Supplement: Supplementary file 7 — Additional file 7. FigS3. GO terms plot of the DEMs, Colors in different plots indicate the level of significance. (A) Biological processes (B) Cellular components (C) Molecular functions. [file 12920_2023_1478_MOESM7_ESM.zip › Additional file 7.jpg]

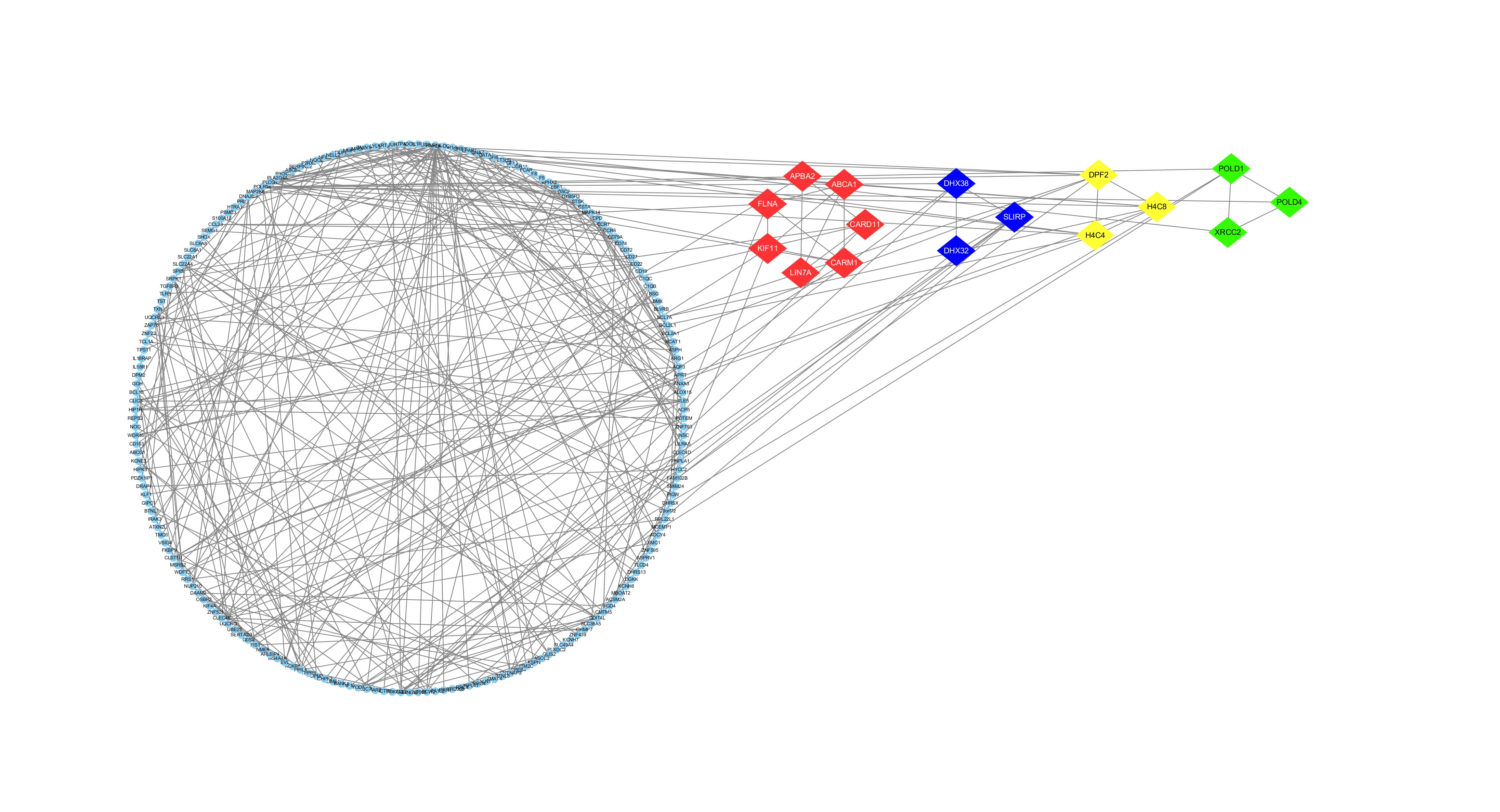

Supplement: Supplementary file 9 — Additional file 9. FigS5. Clusters of the PPI network based on the Metascape and MCODE analysis. Four colors of red, bule, yellow and green indicate four clusters identified by MCODE analysis. [file 12920_2023_1478_MOESM9_ESM.zip › Additional file 9.jpg]

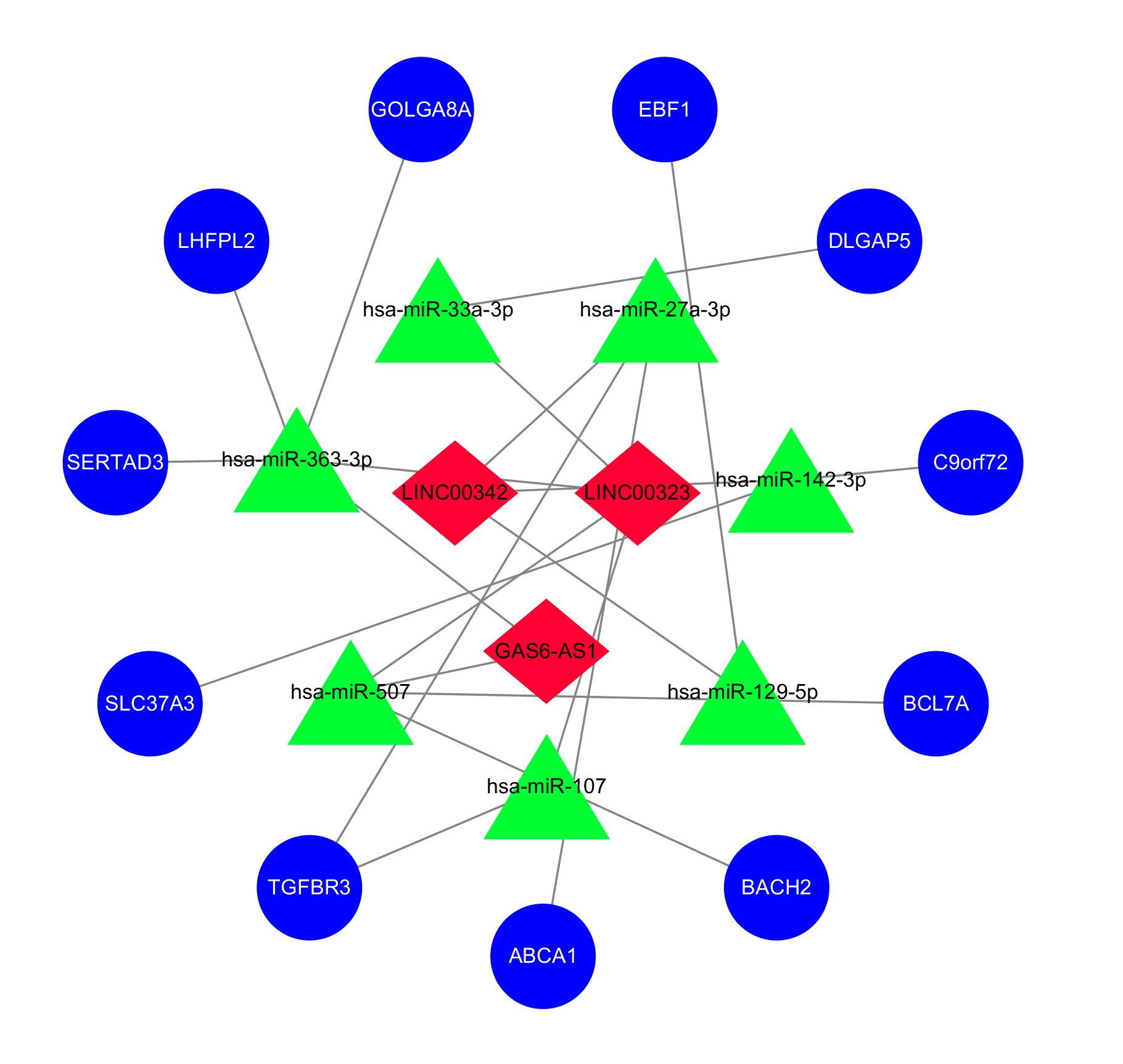

Supplement: Supplementary file 14 — Additional file 14. FigS6. CeRNA regulatory network. Red rhombuses represent lncRNAs, green triangles represent miRNAs and blue circles represent mRNAs, respectively. [file 12920_2023_1478_MOESM14_ESM.zip › Additional file 14.png]

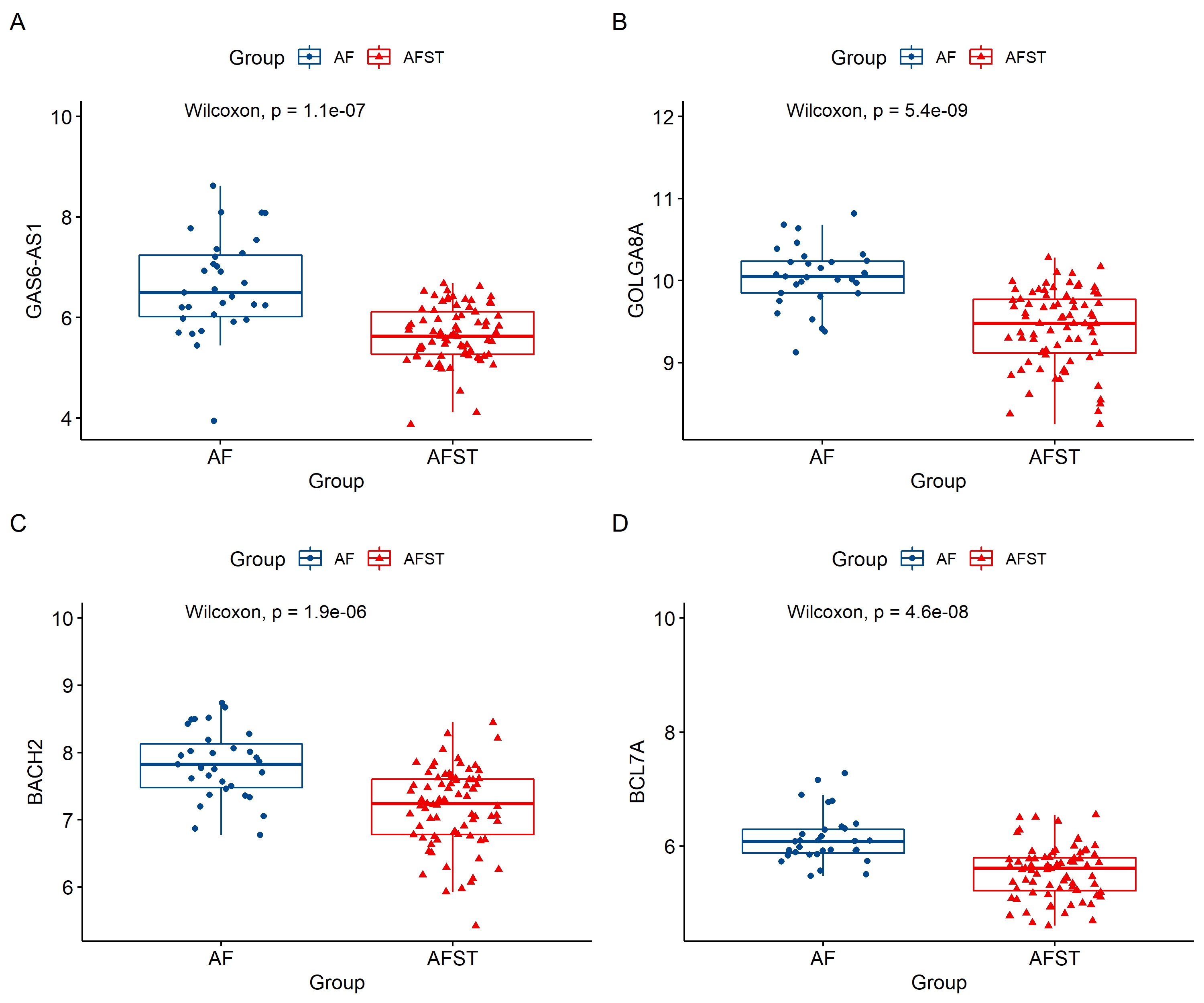

Supplement: Supplementary file 15 — Additional file 15. FigS7. Boxplot of the expression level for four hub genes. [file 12920_2023_1478_MOESM15_ESM.zip › Additional file 15.jpg]

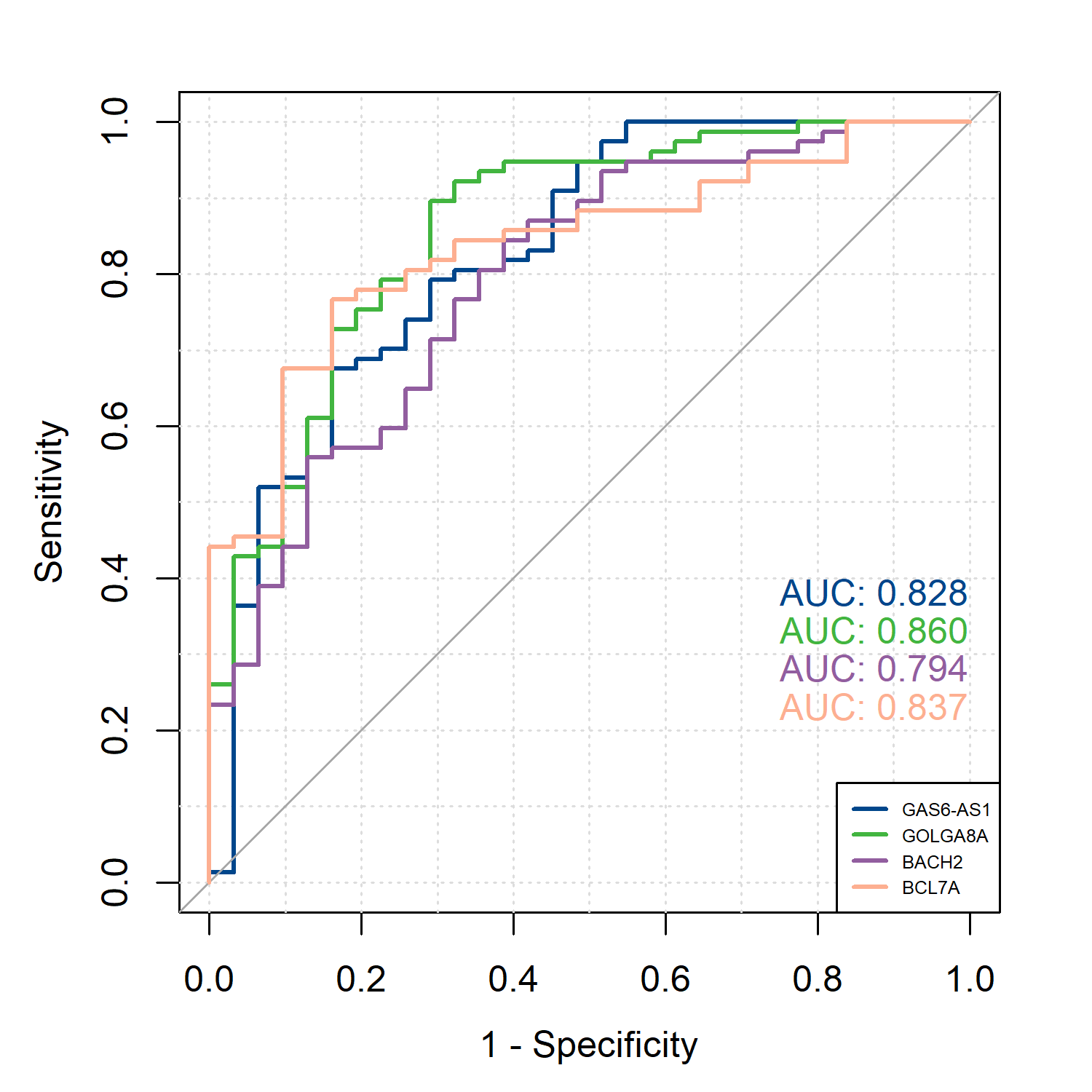

Supplement: Supplementary file 16 — Additional file 16. FigS8. The receiver operator characteristic curves of GAS6-AS1, GOLGA8A, BACH2 and BCL7A for AFST. [file 12920_2023_1478_MOESM16_ESM.zip › Additional file 16.tiff]
