# Supplementary material for: GAS6-AS1, a long noncoding RNA, functions as a key candidate gene in atrial fibrillation related stroke determined by ceRNA network analysis and WGCNA
Source: BMC Med Genomics. 2023 Mar 9;16:51. doi: 10.1186/s12920-023-01478-y (PMC9996875; doi:10.1186/s12920-023-01478-y)
Supplement: Supplementary file 2 — Additional file 2. FigS2. PCA plot of the data before and after the batch effect removal. (A) PCA results before the batch effect removal. (B) PCA results after the batch effect removal. [file 12920_2023_1478_MOESM2_ESM.zip › Additional file 2 legend.docx]

Additional file 2: FigS2 PCA plot of the data before and after the batch effect removal. (A) PCA results before the batch effect removal. (B) PCA results after the batch effect removal.
