# Supplementary material for: GAS6-AS1, a long noncoding RNA, functions as a key candidate gene in atrial fibrillation related stroke determined by ceRNA network analysis and WGCNA
Source: BMC Med Genomics. 2023 Mar 9;16:51. doi: 10.1186/s12920-023-01478-y (PMC9996875; doi:10.1186/s12920-023-01478-y)
Supplement: Supplementary file 4 — Additional file 4. Differentially expressed mRNAs. [file 12920_2023_1478_MOESM4_ESM.zip › Additional file 4 legend.docx]

Additional file 4: Differentially expressed mRNAs
