# Supplementary material for: GAS6-AS1, a long noncoding RNA, functions as a key candidate gene in atrial fibrillation related stroke determined by ceRNA network analysis and WGCNA
Source: BMC Med Genomics. 2023 Mar 9;16:51. doi: 10.1186/s12920-023-01478-y (PMC9996875; doi:10.1186/s12920-023-01478-y)
Supplement: Supplementary file 7 — Additional file 7. FigS3. GO terms plot of the DEMs, Colors in different plots indicate the level of significance. (A) Biological processes (B) Cellular components (C) Molecular functions. [file 12920_2023_1478_MOESM7_ESM.zip › Additional file 7 legend.docx]

Additional file 7: FigS3 GO terms plot of the DEMs, Colors in different plots indicate the level of significance. (A) Biological processes (B) Cellular components (C) Molecular functions
