# Supplementary material for: GAS6-AS1, a long noncoding RNA, functions as a key candidate gene in atrial fibrillation related stroke determined by ceRNA network analysis and WGCNA
Source: BMC Med Genomics. 2023 Mar 9;16:51. doi: 10.1186/s12920-023-01478-y (PMC9996875; doi:10.1186/s12920-023-01478-y)
Supplement: Supplementary file 8 — Additional file 8. FigS4. Circle plot of the GO enrichment analysis. The left outer semicircle represents the logFC value of the genes, and the right semicircle corresponds to GO terms enriched. [file 12920_2023_1478_MOESM8_ESM.zip › Additional file 8 legend.docx]

Additional file 8: FigS4 Circle plot of the GO enrichment analysis. The left outer semicircle represents the logFC value of the genes, and the right semicircle corresponds to GO terms enriched
