# Supplementary material for: GAS6-AS1, a long noncoding RNA, functions as a key candidate gene in atrial fibrillation related stroke determined by ceRNA network analysis and WGCNA
Source: BMC Med Genomics. 2023 Mar 9;16:51. doi: 10.1186/s12920-023-01478-y (PMC9996875; doi:10.1186/s12920-023-01478-y)
Supplement: Supplementary file 9 — Additional file 9. FigS5. Clusters of the PPI network based on the Metascape and MCODE analysis. Four colors of red, bule, yellow and green indicate four clusters identified by MCODE analysis. [file 12920_2023_1478_MOESM9_ESM.zip › Additional file 9 legend.docx]

Additional file 9: FigS5 Clusters of the PPI network based on the Metascape and MCODE analysis. Four colors of red, bule, yellow and green indicate four clusters identified by MCODE analysis.
