# Supplementary material for: GAS6-AS1, a long noncoding RNA, functions as a key candidate gene in atrial fibrillation related stroke determined by ceRNA network analysis and WGCNA
Source: BMC Med Genomics. 2023 Mar 9;16:51. doi: 10.1186/s12920-023-01478-y (PMC9996875; doi:10.1186/s12920-023-01478-y)
Supplement: Supplementary file 13 — Additional file 13. miRNA target mRNA prediction. [file 12920_2023_1478_MOESM13_ESM.zip › Additional file 13 legend.docx]

Additional file 13: miRNA target mRNA prediction
