# Supplementary material for: GAS6-AS1, a long noncoding RNA, functions as a key candidate gene in atrial fibrillation related stroke determined by ceRNA network analysis and WGCNA
Source: BMC Med Genomics. 2023 Mar 9;16:51. doi: 10.1186/s12920-023-01478-y (PMC9996875; doi:10.1186/s12920-023-01478-y)
Supplement: Supplementary file 16 — Additional file 16. FigS8. The receiver operator characteristic curves of GAS6-AS1, GOLGA8A, BACH2 and BCL7A for AFST. [file 12920_2023_1478_MOESM16_ESM.zip › Additional file 16 legend.docx]

Additional file 16: FigS8 The receiver operator characteristic curves of *GAS6-AS1*, *GOLGA8A, BACH2* and *BCL7A* for AFST
